# Acute Neonatal Parotitis with Late-Onset Septic Shock due to *Streptococcus agalactiae*

**DOI:** 10.1155/2014/689678

**Published:** 2014-02-05

**Authors:** M. Boulyana

**Affiliations:** Department of Pediatrics, Hospital of the Saint Omer Region, 62505 Saint Omer, France

## Abstract

Acute neonatal parotitis (ANP) is a very rare disease. Most cases are managed conservatively; early antibiotics and adequate hydration may reduce the need for surgery. The most common cause of ANP is *Staphylococcus aureus*. We report a rare case of acute neonatal parotitis with late-onset septic shock due to *Streptococcus agalactiae*. The diagnosis was confirmed with ultrasound and isolation of *Streptococcus agalactiae* from blood culture. The patient was treated successfully with 10 days of intravenous antibiotics and supportive measures. Despite being rare, streptococcal ANP should be considered in the etiological diagnosis of neonatal sepsis. Early diagnosis and appropriate antibiotic might prevent serious complications.

## 1. Introduction

Acute neonatal parotitis (ANP) is a rare infection with a prevalence of 3.8/10 000 admissions for neonates [1] and an incidence of 13.8 per 10,000 admissions [2]. Only few cases of ANP are reported in literature and the most predominant pathogen is *Staphylococcus aureus* [3]. Here, we report a rare case of ANP with late-onset septic shock due to *Streptococcus agalactiae*.

## 2. Case Report

A 3-week-old girl was admitted to hospital because of irritability and reduced feeding. She was full term, and her birth was a spontaneous vaginal delivery. There was no risk factor for neonatal sepsis (no maternal colonization by Streptococcus group B, no prolonged rupture of membranes). On admission, her weight was 3485 g and she had a high grade fever at 39°C, mottling, and tachycardia at 180 per minute. On examination, a warm and erythematous swelling was noted over the left parotid region. The rest of the physical examination was unremarkable and her fontanelle was normal. Laboratory findings revealed a white blood cell count of 8.5 × 10^9^/L (normal ranges 5–15) with 53% neutrophils, a hemoglobin of 9.5 g/dL (normal 11–16), an elevated plasma procalcitonin of 20 ng/L (normal 0–0,05), an elevated plasma C-reactive protein of 7,5 mg/dL (normal 0–0,3), a hyperlactatemia of 5,6 mmol/L (normal 1–2,8), and a hyperglycemia of 150 mg/dL (normal 60–100). The coagulation, liver, and renal function tests were normal. The blood culture revealed a type III Group B Streptococcus (GBS) or *Streptococcus agalactiae*. No other organisms were identified from other parts of the body or secretions (urine and cerebrospinal fluid cultures were sterile).

**Figure 1:**
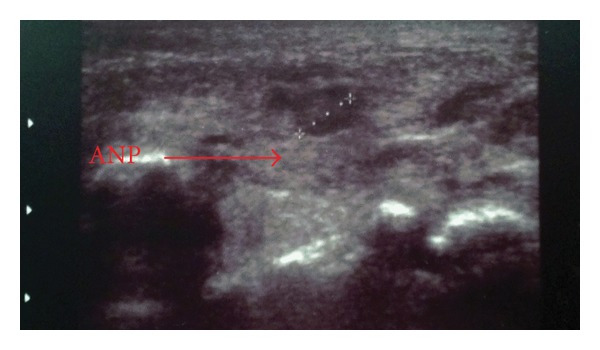
Sonogram of left acute neonatal parotitis (ANP).

Ultrasound revealed enlarged left parotid gland with hypoechoic areas compatible with ANP. No evidence of abscess collection was detected. Also, intraparotid lymph nodes of millimeters in diameter were detected (Figure 1). Differential diagnosis with cellulitis-adenitis syndrome was based on clinical manifestations with supporting ultrasound findings.

Once diagnosed with ANP, our patient was treated with cefotaxime and gentamicin because of severity of septic shock on admission and the late-onset sepsis. After the susceptibility report at day 2, cefotaxime was changed to amoxicillin for 8 more days. After 1 day the fever resolved and on the fourth day of treatment the parotid swelling resolved and the CRP was normalized. A complete evaluation ruled out any immune defect. Examination at followup after 18 months revealed no residues or abnormalities of the gland and she did not show chronic recurrent parotitis.

## 3. Discussion

ANP is a rare infection and tends to occur in immunocompromised patients. The prevalence of acute neonatal suppurative parotitis (NSP) is 3.8/10000 admissions in one report from Italy [1] and an incidence of 13.8 per 10000 admissions [2]. Although bacterial seeding of the parotid can occur hematogenously, infection is more common from oral flora ascending via Stensen's duct in a retrograde fashion into the gland [4, 5]. Spiegel et al. [1] reviewed the cases of patients with ANSP during the past 35 years, mostly from case reports. Common predisposing conditions include dehydration, duct stasis, and immune suppression [4, 6]. Prematurity should be considered as a major risk factor for the infection [1, 4, 6].

The presence of purulent discharge expressed from the opening of the parotid (Stensen's) duct is considered pathognomonic of neonatal suppurative parotitis. ANP was unilateral in most cases. Fever, swelling, and redness of the parotid region are the most prevalent signs [1, 4, 6]. The differential diagnoses for facial swellings in infants include trauma, maxillary infections, lipomas, and adenomas [4]. Laboratory findings are usually nonspecific with a raised white cell with a predominance of neutrophils [2, 5]. The diagnostic criteria for acute NSP are characterized by the triad of parotid swelling, purulent exudate from Stensen's duct, and the growth of pathogenic bacteria in the parotid pus culture [4, 7]. In infants with an unusual clinical presentation, ultrasound examination can help guide the diagnosis and may reveal a diffusely enlarged gland [1, 4, 8]. Examination with ultrasound is noninvasive and useful for diagnosis, differential diagnosis, and excluding the other predisposing factors like anatomical abnormalities of Stensen's duct such as sialectasis, mechanical salivary duct obstruction secondary to a sialolith, and infection related to a parotid gland neoplasm. It can also help determine whether a parotid swelling has arisen secondary to enlargement of adjacent tissue or to the presence of an intraparotid mass, including an abscess [1, 8].

Diagnosis of ANP in our patient was based on clinical signs, ultrasound findings, and the growth of type III Group B Streptococcus or *Streptococcus agalactiae* in blood culture.

*Staphylococcus aureus* is the most commonly cultured organism from neonates with ANP, accounting for approximately 55% of cases [4]. Other organisms include *Streptococcus pyogenes*, *Escherichia coli*, *Klebsiella pneumoniae*, *Pseudomonas aeruginosa*, and anaerobic species [4]. Antibiotics should be chosen to cover this range of potential microbes. A penicillinase-resistant penicillin or first generation cephalosporin to effectively cover *S. aureus* and clindamycin or a similar medication to cover possible anaerobic infection are good initial choices until better direction can be obtained from the study of cultures. A treatment period of 7 to 10 days appears to be adequate [1, 3, 4]. In our case, the empirical therapy by cefotaxime was chosen with gentamicin, either because of the severity of septic shock on admission or because of pathogen associated with late-onset sepsis and ANP. After the susceptibility report at day 2, cefotaxime was changed to amoxicillin for 8 days. Most cases of NSP are managed conservatively with antibiotic therapy [1, 7]. Early antibiotic and advances in antimicrobial therapy reduce the need for surgery and improve prognosis [1, 2, 9]. If prompt clinical improvement does not occur or if the swelling becomes fluctuant, incision and drainage should be performed for abscess formation [2, 4]. Historically, the reported complications of NSP include salivary fistula, facial palsy, deep neck space infections, mediastinitis, and extension of the infection into the external ear, all of which are associated with a poor prognosis [1, 4].

## 4. Conclusion

Despite being rare, ANP should be suspected in newborns with sepsis or facial swelling. Effective treatment involves the prompt antibiotics and adequate hydration. Ultrasound examination may help in the diagnosis.
